# Shaping science: Scholarly motivation and research outcomes in NIH-HEAL funded studies

**DOI:** 10.1371/journal.pone.0343417

**Published:** 2026-03-03

**Authors:** Bryce Kushmerick-McCune, Ginnie Sawyer-Morris, Merve Ulukaya, Kendra Clark, Scott T. Walters, Faye S. Taxman

**Affiliations:** 1 Department of Criminal Justice and Criminology, Sam Houston State University, Huntsville, Texas, United States of America; 2 Friends Research Institute, Baltimore, Maryland, United States of America; 3 Schar School of Policy and Government, George Mason University, Arlington, Virginia, United States of America; 4 College of Public Health, University of North Texas Health Science Center, Fort Worth, Texas, United States of America; Virginia Commonwealth University School of Medicine, UNITED STATES OF AMERICA

## Abstract

In 2018, the National Institutes of Health (NIH) launched the Helping to End Addiction Long-Term ^®^ (HEAL) Initiative to advance addiction and pain research and clinical practice. The NIH-HEAL Initiative supports investigator-initiated research across diverse focus areas, allowing scientists discretion in selecting research topics, methodologies, and outcomes while still remaining subject to NIH’s peer review process. This study aims to answer the research question: *what do investigators of NIH-HEAL funded studies define as important knowledge, particularly in relation to growth in empirical studies and clinical policy?* By examining how pain and addiction researchers define what knowledge is important to produce, this study offers novel insight into how research priorities take shape and why this matters for science and clinical practice. To answer this research question, we conducted a directed content analysis of 1,068 NIH-HEAL funded study abstracts published on the NIH RePORTER website. Our findings suggest that while the scope of the NIH-HEAL funded studies is broad, there are common identifiable patterns that exist within the funded research portfolio such as a preference for treating pain and addiction conditions with prescription medication (33.80% of pain studies, 34.17% of opioid use disorder studies) and a narrow focus on intervention efficacy (43.21% of pain studies, 39.22% of opioid use disorder studies). This analysis is important for the scientific enterprise and clinical practice as prior literature has painted research as inherently influenced by the values of a scientist and of their discipline. By identifying what social and empirical problems NIH-HEAL funded investigators identify, their proposed solutions, and the outcomes they prioritize, this study carries important implications for grant-funding agencies, scientists, and the people who need and receive treatment for pain and addiction conditions.

## Introduction

In 2018, the National Institutes of Health (NIH) launched the Helping to End Addiction Long-Term ^®^ (HEAL) Initiative to advance addiction and pain research and clinical practice. From 2018 to 2023, NIH-HEAL released more than 200 funding announcements and funded 1,072 unique studies [1]. These funding announcements called for proposals in a variety of research areas, including, *1)* Cross-cutting Research, *2)* Enhanced Outcomes for Infants and Children Exposed to Opioids, *3)* New Strategies to Prevent and Treat Opioid Addiction, *4)* Novel Therapeutic Options for Opioid Use Disorder and Overdose, *5)* Preclinical and Translational Research in Pain Management, *6)* Clinical Research in Pain Management, and *7)* Translation of Research to Practice for the Treatment of Opioid Addiction (NIH, n.d.). This $3 billion portfolio includes basic science, preclinical research, clinical trials, implementation studies, and dissemination research [1]. NIH-HEAL operates using an investigator-initiated research model, where applicants are afforded “leeway to generate [their] own ideas and center [their] research within [their] own interests and expertise” [2]. Thus, while NIH-HEAL proposed several research focus areas, investigators can choose what specific research topics to pursue, what questions to ask, what innovations to study, what outcomes to examine, and what methodology to use; including specific request for proposals. However, proposals are subject to NIH’s review process (a scientific peer review and a review by an institute-specific advisory council [3]), which may constrain individual scientist’s discretion by reinforcing disciplinary norms and priorities.

The research-related choices of NIH-HEAL funded investigators is an understudied area. The current study aims to answer the following research question: *What do investigators of NIH-HEAL funded studies define as important knowledge, particularly in relation to growth in empirical studies and clinical policy?* By examining how pain and addiction researchers define what knowledge is important to produce, we offer novel insight into how research priorities take shape and their downstream implications for the scientific enterprise and clinical practice. To answer this research question, three sub-questions were explored. First, *what social/empirical problems do investigators identify and how do they conceptualize these problems*? Second, *what do investigators believe is the path forward in alleviating these social/empirical problems*? Finally, *what are the research outcomes that investigators believe are important to evaluate*? This analysis is important for the scientific enterprise and clinical practice. Prior literature has painted research as inherently influenced by the values of a scientist and of their discipline [4]. Personal values are the beliefs, principles, or priorities held by the individual researcher, which are influenced by ethics, personal interests, or commitments to certain causes [5]. Disciplinary values are the norms, priorities, and assumptions shared by researchers within a particular field of study, including methodological preferences and which research topics are deemed important [5]. When values influence the research process, knowledge production is affected by what researchers and disciplines prioritize, historically without a process for input from the impacted community [6]. Further, the investigator-initiated process creates an environment where the scientists define what they desire to study and the proposal review process reinforces disciplinary norms and priorities, increasing the degree to which values influence research-related choices. This carries implications for investigators, funding agencies, and clinical practice and subsequent policy recommendations. To explore these research questions, we conducted a directed content analysis of 1,068 study abstracts. [As explained further in the methodology section, our sample size is reduced to 1,068 studies (from 1,072) due to missing information from abstracts.] studies noted above.

### Literature review

Scientists pride themselves on their objectivity [4], that is, their ability to pursue value-free and “truthful” knowledge. Indeed, the belief that ethical or social values do not influence research practices or agendas is widely held among the scientific community [7]. However, a growing body of literature questions whether science and scientists are truly objective given their vested interest in certain research questions and topic areas. Intemann and Melo-Martin [7] write, “Science aims not only to discover truths about the world but to ascertain particularly interesting or important truths that can help improve the quality of life for all” (p. 655). While choices are constrained by disciplinary norms, peer reviewers, and grant-funding agencies, scientists generally enjoy agency in selecting what problems to study [8,9].

The selection of issues to be studied lays – in large part – on the values held by the scientists. Values are shaped by personal interests, societal institutions, governmental organizations, grant-funding agencies, and the disciplines scientists are operating within [5,10,11]. The role of disciplines in shaping knowledge affects objectivity since the discipline-specific questions may differ from multi-disciplinary research questions. Reflecting on this, Biber [5] writes, “The collective imprimatur of scientists in a field is what ultimately determines what is “knowledge” and “truth” within the field, what work is valid, which scientists should be praised” (p. 509) – suggesting that scientists’ work is affected by the environment and is not always value-free. The selection of research topics is also influenced by a researcher’s prior work, identified gaps in the literature, and the perceived knowledge needs of the public and policymakers [12,13].

Often, scientists do not necessarily reflect on or recognize how their own personal values and experiences influence their research decisions [11]. Yet, this is important to consider given the influence of values on the research process and knowledge production. Values shape the identification and conceptualization of scientific problems and study outcomes [4], particularly in trying to understand or address what scientists believe is an essential social goal [5]. Intemann and Melo-Martin [7] outline how values influence every aspect of the research process, detailing how values influence what constitutes an important area of study, how research questions should be framed, what research projects should be funded, and what methodology(s) should be used. Additionally, scientists are often motivated to conduct “research of consequence” [14] that can be used to shape policy [15], including clinical practice, particularly when a discipline sees itself as responding to a “crisis” [5].

Considering how a researcher’s own values and the disciplinary norms under which they operate shape motivations, research questions, processes, and outcomes is important for both theory and practice. With value-influenced research, necessary policies could be misguided or, in some instances, nonexistent given a lack of scientific attention to a topic or phenomenon. Theoretically, with value-influenced research, knowledge production is limited to what researchers and disciplines believe is important. Collectively, this literature underscores the theoretical and practical importance of understanding how scientists define important knowledge and research outcomes. Given the focus of this paper on how pain and addiction scholars conceptualize and explore research problems, below, we review what the existing research has discovered about this topic.

### Addiction and pain research trends

One way to understand how researchers conceptualize social problems and formulate research questions is to examine publication trends. Many researchers use bibliometric analysis, a statistical method for analyzing study patterns in publications [16]. Interestingly, while there are a large number of topic-specific bibliometric analyses for pain and addiction studies (e.g., genetic predictors of addiction or post-surgical pain [17,18]), there are fewer discipline-wide studies of pain and addiction research that examine publication trends over a wide range of topic areas. Of the discipline-wide studies, more look at addiction research than pain research – limiting a holistic understanding of pain research publication trends. Below, we review what bibliometric analyses have revealed about trends in pain and addiction research.

#### Pain research.

Arendt-Nielsen and colleagues [19] analyzed pain-related publications from the European Journal of Pain and PAIN databases and found that the focus of pain studies have changed over time from mono-disciplinary studies (like behavioral studies) to multi-disciplinary studies (like combined behavioral and cell studies). Most pain-publication bibliometric analyses focus on specific topics, like the treatment of cancer pain with opioids [20], low back pain treatment [21], post-surgical pain [17], and neuropathic pain [22], as examples. One study [23] referenced the relationship between opioid prescription and addiction, which is pertinent to the NIH-HEAL initiative. The authors examined publication trends relating to this topic, particularly how researchers responded to Portenoy and Foley’s [24] article supporting the use of opioids for pain management. In their analysis, the authors identify three research time periods over roughly three decades from 1986 to 2019: exploration (i.e., studying the effects of opioids), implementation (i.e., studying/advocating the use of opioids), and reassessment (i.e., recognizing the harms of opioids). From these focused analyses, we see a wide breadth of pain-related research topics, highlighting how researcher values appear to shape research interests.

#### Addiction research.

Bibliometric analyses of addiction research papers have produced key themes. First, the United States leads in producing addiction science [25–27]. Moreover, this research is overwhelmingly funded by the United States Department of Health and Human Services, specifically the National Institutes of Health [26,27]. Second, there are a variety of research focus areas that scientists pursue. Martin del Rio and colleagues [25] examined the top 100 most-cited substance abuse papers and found that authors most often examined evaluation/diagnosis of addiction, with other common research topics including determinants of addiction and evaluation of interventions. Valderrama Zurian and colleagues [26] conducted a more focused analysis looking only at research pertaining to cannabis, heroin, cocaine, and psychostimulants and found that the most cited works examined the neurobiology of addiction, illustrating a disciplinary focus on the relationship between physiology and addiction. Looking at the drug abuse research published by the top 10-producing countries, Varmazyar and colleagues [27] revealed that most drug abuse studies focused on psychiatry, followed closely by substance use and neuroscience. Further analysis revealed four distinct research focus areas examined by drug abuse scholars – alcohol, cocaine, opioids, and cannabis. Scholars studying opioids were most commonly interested in abuse, medication-assisted treatment, pain, and overdose. Other research finds that severity, quality of life, and mental health indicators are common outcomes pursued by addiction scholars [28]. Finally, other bibliometric analyses focus on specific topics, like prescription opioid deaths [29], social connectedness [30], genetic predictors of addiction [18], and neonatal abstinence syndrome [31].

### Research gap and study significance

While the above referenced research helps to ascertain an understanding of how pain and addiction scholars define important knowledge, there are gaps in knowledge that this study will address. First, prior research is limited because it analyzed pre-determined themes/topics (e.g., prescription opioid deaths [29]). Directed content analysis, which we use in this study, allows for relevant research findings or theory to guide initial code development [32]. In this case, we focused on investigator motivations and outcomes because they indicate what topic areas scientists view as important. Subsequent coding – that is, all coding that occurred after establishing a primary code list before coding began – was conducted with an inductive approach that allows the data to speak for itself [33]. Said another way, by taking an inductive approach, we can ascertain themes that emerge *from* the data, not through a pre-determined theme list. Second, we provide an in-depth examination of pain and addiction study abstracts funded by NIH, one of the leading grant-funding agencies, offering a closer look at not only what researchers choose to study, but also what topics reviewers deem as important and what studies NIH-HEAL was interested in funding at the time. Finally, we examine project abstracts, not published papers. Research proposals may better provide a more complete picture of what a researcher plans to study, as published papers often only report on fragments of the research. This provides a unique opportunity to examine planned, funded studies that will generate the next generation of findings, not just published papers.

## Materials and methods

The data presented here comes from a larger landscape analysis of NIH-HEAL funded studies, which was aimed at understanding opportunities for knowledge-building, transfer, and utilization as well as translational gaps. To identify the sample for the landscape analysis, a review of NIH administrative data was conducted. All abstracts (N = 1,526) were reviewed by two project managers and after excluding competitive renewals, grant supplements with duplicate titles and abstracts, and pilot work for biphasic awards, a core sample of 1,072 HEAL projects were selected for analysis. The application IDs for the projects were used to query the NIH RePORTER website [34] to identify published abstracts, concept papers, and protocol papers funded by HEAL during FY18-FY2023. NIH RePORTER is a Congressionally mandated tool to improve the transparency of science. The abstracts (and additional protocol papers if they were available) served as the primary data source for the landscape analysis. All study protocols were deemed exempt by George Mason University’s Institutional Review Board.

To conduct the landscape analysis, two members of the research team and the project lead (PI Taxman) developed and refined a codebook based on Consolidated Framework for Implementation Research (CFIR) domains (e.g., nature of the innovation studied, outer setting, inner setting, change processes, and characteristics of the individuals involved), coding guidelines, and relevant literature [35,36]. The CFIR framework was selected because it provides an overview of the key domains that affect under what circumstances an innovation is implemented. This codebook informed the development of a 54-item data collection tool designed in Qualtrics. A team of researchers was extensively trained on how to use this tool, and researchers did not begin coding independently until they attained 90% consensus with the trainers. Data collection occurred from June 2023 till August 2024, and trainers held monthly meetings to share updates, obtain feedback about the tool, and address emerging questions. Data was exported from Qualtrics and cleaned in Stata version 18.0.

### The current study

The main objective of the current study was to answer the research question, “*What do investigators of NIH-HEAL funded studies define as important knowledge, particularly in relation to growth in empirical studies and clinical policy?”.* This study utilized a directed content analysis approach to explore two components of the abstracts in more depth, *1)* innovation outcomes and *2)* researcher interests including motivation for the study. As noted, 1,072 NIH-HEAL funded abstracts were identified for the larger project. However, only 1,068 abstracts are included in this analysis because the NIH RePORTER website did not display information for four abstracts. This content analysis considered responses to two items on the 54-item data collection tool. The study outcome question was “*What are the primary outcomes under investigation?*” and the motivation question was “*State the problem that is being addressed and identify the rationale for addressing the study?*”.

After the first round of coding (described above), study outcomes and motivations were downloaded into separate Microsoft Word documents and linked to the qualitative data analysis program Atlas.ti. The lead author independently reviewed and coded the scripts. Outcome and motivation scripts were coded using a directed content analysis approach, where researchers quantify and analyze the presence of themes or concepts [37]. A directed content analysis allows for relevant research findings, theory, or existing data (in this case, outcomes and motivations) to guide initial code development [32]. When scripts were missing a substantial amount of information (e.g., simply, “analgesia” or “improving methadone treatment”), the study abstract was reviewed on the NIH RePORTER website. Two team members, the lead author and a doctoral-level project manager, met multiple times to test the themes and map them onto the CFIR domains. All members of the research team reviewed and discussed the coding results.

In total, 711 codes were inductively derived from the data. Outcome coding produced a total of 506 individual codes. Abstracts were often assigned multiple outcome codes with an average of five outcome codes applied to each abstract. Motivation coding produced a total of 205 individual codes. Abstracts were often assigned multiple motivation codes with an average of four motivation codes applied to each abstract. To answer the primary research question, “*What do investigators of NIH-HEAL funded studies define as important knowledge, particularly in relation to growth in empirical studies and clinical policy?”* we organized our analysis into three sections. First, we examined how NIH-HEAL investigators identify and conceptualize social problems. Second, we reviewed the solutions or study areas that authors note are necessary to alleviate these identified problems. Finally, we explore the different ways that authors evaluate their proposed innovations. In Table 1 below, we present the codes used to explore these three questions.

**Table 1:** Code groups for outcome and motivation coding.

Code Group	Definition
***Identifying/Conceptualizing Problems (n = 232 total codes, 4 sub-themes)***	
Identified Problems (77 unique codes)	Identified social and/or clinical practice problems
Gaps in the Existing Research (55 unique codes)	Identified knowledge/practice gaps in the literature
Conceptualizing Addiction (64 unique codes)	How authors describe addiction
Conceptualizing Pain (36 unique codes)	How authors describe pain
	
***Charting a Path Forward (n = 230 total codes, 9 sub-themes)***	
Research and Practice Needs (73 unique codes)	Proposed solutions to alleviate existing problems.
OUD Tx (42 unique codes)	How authors propose treating OUD
SUD Tx (23 unique codes)	How authors propose treating SUD
Pain Tx (39 unique codes)	How authors propose treating pain
Other Medical Conditions (OMC) Tx (14 unique codes)	How authors propose treating additional conditions
Maternal and Infant Health (14 unique codes)	Focuses on opioid-related maternal and infant health
Addiction Predictors (12 unique codes)	Predictors of addiction
Pain Predictors (10 unique codes)	Predictors of pain
Condition Predictors (3 unique codes)	Predictors of non-pain/addiction conditions
	
***Evaluating Solutions (n = 249 total codes, 4 sub-themes)***	
OUD Tx Evaluation (112 unique codes)	How authors evaluate OUD treatment innovations
SUD Tx Evaluation (31 unique codes)	How authors evaluate SUD treatment innovations
Pain Tx Evaluation (89 unique codes)	How authors evaluate pain treatment innovations
OMC Tx Evaluation (17 unique codes)	How authors propose evaluating these treatment innovations

*Tx = Treatment.*

## Results

### Identifying and conceptualizing problems

This section describes the different social problems that investigators identified as well as how they conceptualize these problems. Researchers identified 77 different problems to address. As displayed below in Table 2, frequently identified problems include *1)* issues with existing pain treatment strategies, *2)* addiction resulting from pain treatment, *3)* drug overdose, *4)* OUD generally, *5)* impacts for special populations, and *6)* need for supplemental grants. The remaining 71 problems were identified by less than 10% of investigators but ran the gamut from describing how pain decreases quality of life to mental health to issues with access to addiction or pain treatment.

**Table 2:** Frequently identified social problems.

Identified Problem	Number of Studies
Issues with existing pain treatment	199 (18.63%)
Addiction resulting from pain treatment	130 (65.33%*)
Drug overdose	163 (15.26%)
OUD generally	154 (14.42%)
Impacts for Special Populations	136 (12.73%)
Supplemental Grants	133 (12.45%)

*Addiction resulting from pain treatment was a specific issue identified with existing pain treatment. Thus, this percentage reflects how many of the 199 studies noting issues with existing pain treatment specifically discussed addiction resulting from pain treatment (i.e., (130/199)*100 = 65.33%).

The most identified problem included issues with existing pain treatment strategies (n = 199, 18.63%). Predominantly, these authors focused on addiction resulting from pain treatment. Of those 199 abstracts, 65.33% (n = 130) mentioned this issue. For example, one author wrote “The expansion of opioid prescribing in recent years to better treat pain has markedly increased their usage and availability and fueled an epidemic of abuse. Estimates of up to 80% of addicts reported initiating their habit through prescription drugs” (PI: Ying-Xian Pan; 1UG3DA048379−01). Other authors who noted problems resulting from pain treatment talked about additional side effects like “tolerance, constipation, respiratory depression, physical dependence and high addictive potential” (PI: Ajay S. Yekkirala; 2R44DA041912−03) or “a considerable risk of liver toxicity or kidney toxicity” (PI: Robert Naismith; 2SB1NS119103−04). Another commonly referenced issue included drug overdose deaths (n = 163, 15.26%). For example, one researcher commented that in 2016 “2.1 million people met diagnostic criteria for opioid use disorder (OUD). In that same year, 42,249 Americans died of opioid overdose – an average of 116 preventable deaths per day” (PI: Jeffrey Lebrun; 1R44DA049629−01) or “A key challenge of the epidemic is that overdose victims often die because they are alone or among untrained or impaired bystanders and thus do not receive timely resuscitation” (PI: Nicholas M. Mark; 1R44DA050339−01). Researchers frequently studied opioid use disorder generally (n = 154, 14.42%), writing phrases such as “the development of opioid use disorder (OUD) have reached crisis levels in the United States” (PI: Kathryn A. Cunningham; 1UG3DA050317−01) or “OUD has become a public health emergency” (PI: Daniel R. Burnett; 1R43DA049623−01**)**. Researchers also noted how different populations were impacted by a variety of issues (n = 136, 12.73%), like “The highest concentration of opioid users is in the criminal justice system” (PI: Faye S. Taxman; 5U2CDA050097−02) or “Due to cultural stigma attached to breast cancer, cultural stoicism toward pain and symptoms, and language barriers, Asian American breast cancer survivors tend to suffer unnecessarily from pain that could be easily managed” (PI: Eun-Ok Im; 1R61CA280979−01). Finally, many researchers proposed the research as a supplement (add-on) to prior grant work (n = 133, 12.45%), typically to gather new data or apply the innovation to a new population; supplements are common at NIH (and HEAL) to allow researchers to explore new ideas.

Beyond these identified problems, scholars identified 55 gaps in the existing literature. As displayed below in Table 3, frequently identified gaps include *1)* genetic pathways for pain/addiction, *2)* Long-term effects of conditions, and *3)* Unknown treatment options. The remaining 52 research gaps were identified by less than 5% of investigators but included gaps like unknown risk/protective factors, differences in efficacy by population, or how to identify or monitor the condition.

**Table 3:** Frequently identified research gaps.

Identified Gaps	Number of Studies
Genetic pathways for pain/addiction	174 (16.29%)
Long-term effects of conditions	75 (7.02%)
Unknown treatment options	67 (6.27%)

Overwhelmingly, authors (n = 174, 16.29%) highlighted unclear or lacking knowledge about genetic pathways for various pain and addiction issues. For instance, one pain study abstract stated, “fundamental knowledge regarding causes of co-occurrence of multiple chronic pain conditions and neurophysiologic alterations in pain processing is lacking” (PI: Tuhina Neogi; 5R01NS121419−03) and one OUD study abstract highlighted how “The contribution of this proposal is significant because it uncovers a previously unknown mechanism underlying opioid tolerance and provides a promising therapeutic target in the long-term relief of tolerance” (PI: Lingyong Li; 7R01DA056673−02). Other scholars (n = 75, 7.02%) noted that the long-term effects of specific conditions were unknown. Investigators focused on this gap were often researching in-utero exposure to opioids and other drugs. For instance, one of the studies stated, “Preliminary studies show that prenatal opioid exposure is associated with increased risk of impaired neurodevelopment across cognitive, motor and behavioral domains. Therefore, studies to rigorously characterize the impacts of prenatal opioid exposure on early brain development are critically needed” (PI: Stephanie L. Merhar; 1R34DA050268−01). Additional studies (n = 67, 6.27%) explained that preventative or treatment options for various conditions are unclear or unknown. For example, one study focused on the treatment of OUD stated, “there are no established evidence-based interventions that specifically focus on improving MOUD adherence and retention” (PI: Marc Fishman; 1R34DA057627−01).

After identifying these social problems and research gaps, we explored the question of how investigators conceptualize addiction and pain. The thematic coding revealed that addiction is conceptualized in 64 different ways, sorted into five broader categories: *impacts on wellness, indications of addiction, stages of addiction, readiness for treatment*, and *critical areas in the field*. *Impacts on wellness* includes the impact of addiction on mental, physical, and social health, with 29 unique codes. Within the mental health domain of addiction, topics included anxiety, depression, and suicidality (51 mentions). Within the physical health domain, researchers primarily focused on the impact of addiction on sleep (17 mentions) and mortality (13 mentions), and physical health generally (6 mentions). Within the social health domain, researchers predominately noted the social stigma substance-addicted people encountered (14 mentions). *Indications of addiction* includes the different ways in which researchers describe the symptoms of addiction and includes 20 unique codes. The most common way of describing addiction symptoms was overdose (91 mentions), followed by craving (26 mentions), and general substance misuse (22 mentions). *Stages of addiction* includes the different ways in which investigators describe the addiction process and includes seven unique codes, including reducing use (3 mentions), detox (3 mentions), withdrawal (31 mentions), relapse (33 mentions), and recovery (7 mentions). *Readiness for treatment* includes the different ways that researchers describe a person’s perception of addiction treatment and includes five unique codes. Researchers often cited the utilization/accessibility of treatment (33 mentions) and adherence to treatment (31 mentions). Finally, *critical areas in the field* refers to trends in drug use across the country (3 mentions), practitioner drug misuse/mis-prescribing (1 mention) and bioterrorism (1 mention).

Researchers conceptualize pain in 36 different ways, sorted into three broader categories: *impacts on wellness, measurements of pain,* and *readiness for treatment*. *Impacts on wellness* includes the impact of pain on mental, physical, and social health with 14 unique codes. Within the mental health domain, researchers focused on the impact of pain on quality of life (10 mentions), post-traumatic stress disorder (2 mentions), depression (10 mentions), and anxiety (7 mentions). Within the physical health domain, the emphasis was on the impact of pain on opioid use (8 mentions), fatigue (1 mention), and sleep (7 mentions). For the social health domain, researchers focused on the social stigma people in pain received (6 mentions), societal isolation (3 mentions) and employment (2 mentions). *Measurements of pain* includes the different ways in which investigators report measuring pain and includes 18 unique codes including pain severity (23 mentions) and interference (23 mentions). Finally, *readiness for treatment* includes the different ways in which researchers describe a person’s perception of pain treatment and includes four unique codes with utilization and accessibility of pain treatment most frequently mentioned (5 mentions).

### Charting a path forward to advance science and practice

Researchers identified 73 different research and practice needs. These needs are distinct from the social problems identified above in that they reflect solutions or study areas that authors note are necessary to alleviate existing problems. As displayed in Table 4, frequently identified needs include *1)* improved addiction treatment, *2)* improved pain treatment, *3)* validation of therapeutic pain targets, *4)* validation of therapeutic addiction targets, *5)* nonaddictive pain control, and *6)* evaluation of existing treatments. The remaining 65 research and practice needs were identified by less than 4% of researchers but covered topics such as dissemination, holistic treatment approaches, and improved diagnostic tools.

**Table 4:** Frequently identified needs.

Identified Needs	Number of Studies
Improved addiction treatment	312 (29.21%)
Improved pain treatment	230 (21.54%)
Validation of therapeutic pain targets	160 (14.98%)
Validation of therapeutic addiction targets	50 (4.68%)
Nonaddictive pain control	78 (7.30%)
Evaluation of existing treatments	59 (5.52%)

As expected, improved addiction treatment (n = 312, 29.21%) and improved pain treatment (n = 230, 21.54%) are primary identified needs. For example, one investigator remarked “fewer than 20% of patients with OUD receive those treatments. We do not have a functioning system to treat OUD. Innovation to the way that we deliver OUD treatment could provide the OUD care we now need” (PI: Benjamin P. Linas; 1R01DA046527−01). Further, 14.98% (n = 160) of investigators stated that research is necessary in validating therapeutic pain targets, while a smaller number of scholars (n = 50, 4.68%) advocated for validating therapeutic addiction targets. For instance, one pain study abstract stated that “Despite the clinical need and the available technology, no comprehensive study has integrated these ultrasound measures to validate a biomarker for the myofascial component of chronic low back pain” (PI: Ajay D. Wasan; 1R61AT012282−01). Other investigators specified that nonaddictive pain control is necessary (n = 78, 7.30%), making comments such as “there is an urgent need for non-addictive pain treatments” (PI: Donald K. Shin; 1R41DA053011−01). Some researchers (n = 59, 5.52%) noted that there was a pressing need to evaluate already existing treatment strategies. For example, one investigator commented, “Mindfulness-based Stress Reduction is now recommended… for initial treatment of [chronic back pain]. The next necessary step is to do a [clinical trial] with the goal of informing decision makers how this program can work in a real-life clinical setting” (PI: Natalia E. Morone; 4UH3AT010621−02).

After specifying problems and needs, investigators proposed a plethora of potential treatments and innovations. Across the 1,068 studies, researchers proposed a total of 114 innovations. These innovations are sorted into four broader categories: OUD treatment (41 innovations), SUD treatment (22 innovations), pain treatment (38 innovations), and other medical conditions treatment (13 innovations). Below, Table 5 displays the most frequently identified innovations within these broader categories.

**Table 5:** Frequently identified innovations.

Identified Innovations	Number of Studies*
***OUD Treatment (n = 357)***	
Prescription drug	122 (34.17%)
Behavioral therapy	39 (10.92%)
Imaging	37 (10.36%)
***SUD Treatment (n = 77)***	
Best practices	13 (16.88%)
Prescription drugs	12 (15.58%)
Case management	10 (12.99%)
Drug tests/screening	8 (10.39%)
***Pain Treatment (n = 361)***	
Prescription drug	122 (33.80%)
Behavioral therapy	32 (8.86%)
Best practices	24 (6.65%)
Electrical stimulation	22 (6.09%)
***Other Medical Conditions Treatment (n = 31)***	
Observation	6 (19.35%)
Suicide prevention	5 (16.13%)

*Percentage reflects the number of investigators who focused on a given innovation within each sub-group (e.g., 34.17% of the 357 OUD treatment studies focused on prescription drugs).

357 of the studies (33.43% of the total sample) were interested in OUD treatment and described 41 ways of treating OUD. Of those 357 studies, the most referenced OUD treatment method was a prescription drug (n = 122, 34.17%) such as Buprenorphine or extended-release Naltrexone. Behavioral therapy was commonly studied (n = 39, 10.92%) as was imaging (n = 37, 10.36%). The remaining 38 OUD treatment methods were mentioned by less than 10% of investigators, but included methods like case management, electrical stimulation, and yoga. 77 investigators (or 7.21% of the total sample) were interested in SUD treatment (additional drugs beyond opioids) and described 22 ways of treating SUD. Of those 77 studies, the most common SUD treatment was developing best practices (n = 13, 16.88%). For example, one SUD study investigator commented, “This work will provide foundational data to develop practical harm reduction delivery strategies for rural areas facing disparate challenges” (PI: Mai Tuyet Pho; 1R01DA057665−01). Prescription drugs were commonly studied (n = 12, 15.58%), as was case management (n = 10, 12.99%) and drug tests/screening (n = 8, 10.39%). The remaining 18 SUD treatment methods were mentioned by less than 10% of investigators but included treatments like behavioral therapy, practitioner training, and mindfulness. 361 investigators (33.80% of the total sample) were interested in pain treatment and described 38 ways of treating pain. Of those 361 studies, the majority focused on evaluating prescription drugs (n = 122, 33.80%). Behavioral therapy was also studied (n = 32, 8.86%) as were best practices (n = 24, 6.65%) and electrical stimulation (n = 22, 6.09%). The remaining 34 pain treatment methods were mentioned by less than 5% of investigators, but included methods like physical therapy, wearable devices, and acupuncture. Finally, 31 investigators (or 2.90% of the total sample) were interested in other medical conditions (e.g., Alzheimer’s, HIV) and described 13 ways of treating these conditions. Of those 31 studies, the most common treatment included observation (n = 6, 19.35%), where investigators collected and analyzed typically genetic data (i.e., no formal treatment). Suicide prevention was also commonly studied (n = 5, 16.13%). The remaining 11 methods were mentioned by less than 10% of investigators, and included methods such as behavioral therapy, vaccines, and wearable devices.

Notably, there were 280 studies that also evaluated various predictors of addiction and pain. Below, Table 6 displays the most frequently identified addiction and pain predictors.

**Table 6:** Frequently identified predictors.

Identified Predictors	Number of Studies
***Addiction Predictors (n = 76)***	
Genetic predictors	37 (48.68%)
Social predictors	21 (27.63%)
Sleep predictors	7 (9.21%)
***Pain Predictors (n = 204)***	
Genetic predictors	189 (92.65%)
Social predictors	12 (5.88%)

76 studies (or 7.12% of the total sample) examined addiction predictors. These researchers describe 11 different predictors. Those 76 studies focused predominantly on genetic predictors (n = 37, 48.68%), remarking, for example, “To identify functional markers expressed in the MOR-expressing neurons that specifically mediate [opioid-induced respiratory depression]” (PI: Sung Han; 1R01DA056658−01) and “whether blocking Tiam1 mediated synaptic plasticity with Tiam 1 inhibitor or antisense oligonucleotides (ASOs) produces the long lasting relief of opioid tolerance” (PI: Lingyong Li; 1R01DA056673-01).” Additional addiction predictors include social predictors (n = 21, 27.63%) and the role of sleep in addictive behavior (n = 7, 9.21%). The remaining eight addiction predictors were mentioned by less than 5% of investigators. 204 studies (19.10% of the total sample) examined pain predictors, and investigators describe nine different predictors. Of those 204 studies, authors overwhelmingly focused on genetic predictors (n = 189, 92.65%), commenting things such as “Determine if sympathetic activation triggers cluster firing of the DRG neurons after peripheral nerve injury” (PI: Xinzhong Dong; 1RF1NS113883−01) and “We will apply “Raman2Omics” to systematically investigate genes and cells associated with postoperative pain at scale” (PI: Jian Shu; 1DP2TR004354−01). Some authors also focused on social predictors (n = 12, 5.88%). The remaining seven pain predictors were mentioned by less than 5% of investigators.

### Evaluating solutions

This section describes the different methods and strategies that investigators use to evaluate their proposed treatments and innovations. Table 7 displays the most frequently identified outcomes for each research sub-group.

**Table 7:** Frequently identified research outcomes.

Identified Innovations	Number of Studies*
***OUD Treatment Evaluation (n = 357)***	
Efficacy	140 (39.22%)
Safety	45 (12.61%)
Impact on brain functioning	44 (12.32%)
Implementation	39 (10.92%)
***SUD Treatment Evaluation (n = 77)***	
Efficacy	27 (35.06%)
Implementation	11 (14.29%)
***Pain Treatment Evaluation (n = 361)***	
Efficacy	156 (43.21%)
Safety	49 (13.57%)
Pain level (*as efficacy-specific outcome*)	43 (11.91%)
***Other Medical Conditions Treatment Evaluation (n = 31)***	
Efficacy	6 (19.35%)
Implementation	4 (12.90%)
Condition predictors	4 (12.90%)

*Percentage reflects the number of investigators who focused on a given outcome within each sub-group (e.g., 39.22% of the 357 OUD treatment studies focused on efficacy)

Researchers describe 112 different evaluation outcomes for OUD treatments. Of the 357 OUD-related studies, most researchers are concerned with the efficacy of their treatment (n = 140, 39.22%)[As a point of clarification, while it is expected that all investigators are concerned with efficacy, only 140 explicitly stated that they are examining efficacy], commenting things such as “Verify that semaglutide is safe and effective in reducing cue/drug/stress-induced heroin seeking” (PI: Patricia S. Grigson; 1UG3DA050325−01) and “The goal of this research proposal is to evaluate the safety and efficacy of brexpiprazole as an adjunctive treatment to buprenorphine maintenance therapy in patients diagnosed with opioid use disorder” (PI: Andy Forbes; 1UG3DA051383-01A1). Other commonly mentioned outcomes include safety (n = 45, 12.61%), the impact of the treatment on brain functioning (n = 44, 12.32%), and implementation (n = 39, 10.92%). The remaining 108 outcomes were mentioned by less than 10% of investigators, and included outcomes such as user adherence, mental well-being, and patient satisfaction. Investigators describe 32 ways of evaluating SUD treatments. Of the 77 SUD-related studies, most authors investigated efficacy (n = 27, 35.06%). Some studies focused on implementation (n = 11, 14.29%), like one that read “Implementation outcomes include implementation process fidelity… staff acceptance of harm reduction philosophies… OFR fidelity to CDC best practices… and usability of the Overdose Touchpoint Dashboard” (PI: Matthew Aalsma; 1R61DA057660−01). The remaining 30 outcomes were mentioned by less than 10% of investigators, and included outcomes such as user engagement, safety, and convenience.

Researchers describe 89 techniques to evaluate pain treatments. Of the 361 pain-related studies, most authors examined efficacy (n = 156, 43.21%). Others examined safety (n = 49, 13.57%), and others specified pain level as their efficacy outcome (n = 43, 11.91%). The remaining 86 outcomes were mentioned by less than 10% of investigators, and included topics such as feasibility, abuse liability, and quality of life. Finally, investigators describe 17 ways of evaluating other medical condition treatments. Of these 31 studies, most investigators focused on efficacy (n = 6, 19.35%), while others focused on implementation (n = 4, 12.90%) or predictors of the condition (n = 4, 12.90%). The remaining 14 outcomes were mentioned by less than 10% of investigators, and included outcomes such as frequency of utilization, sleep patterns, and mental health outcomes.

## Discussion

This study examined how pain and addiction researchers define what knowledge is important to produce. Our findings reveal how research priorities take shape at the funding stage and the implications of these priorities for future scientific and clinical work. Using a directed content analysis approach to analyze 1,068 NIH-HEAL funded study abstracts, we sought to answer the research question: *What do investigators of NIH-HEAL funded studies define as important knowledge, particularly in relation to growth in empirical studies and clinical policy?* We found that authors most often identified issues with existing pain treatments as an important social problem – moreover, they underscored the link between addictive pain medication and substance abuse. Researchers focused on drug overdose and often characterized OUD as a “public health emergency”. Some focused on how social problems particularly impact special populations like racial/ethnic minorities and incarcerated people. Others identified problems centered around gaps in the existing literature on topics such as pain and addiction genetic pathways, long-term effects of conditions, and unclear or unknown treatment options. Finally, investigators conceptualize addiction and pain in interesting ways, including its impacts on wellness, indications and stages of addiction/pain, and readiness for treatment.

Looking at proposed solutions and study areas, improved addiction and pain treatment were (expectantly) primary focuses, though validating therapeutic pain/addiction targets, a need for nonaddictive pain control, and an evaluation of pre-existing treatment strategies were also explored. Researchers focused primarily on evaluating prescription drugs, and mentioned alternative innovations (e.g., behavioral therapy) much less frequently – though, notably, there was an impressive amount of variation in proposed innovations. Finally, examining evaluation outcomes, investigators were predominantly focused on evaluating the efficacy of their proposed innovation, with less attention paid to other outcomes such as implementation and safety. For studies that looked at predictors of pain and addiction, most investigators focused on identifying genetic predictors over alternative predictors like socioenvironmental influences. Collectively, our findings suggest that while the scope of NIH-HEAL funded studies is broad, identifiable patterns exist within the funded research.

### Contribution to theory

The above research contributes to the existing literature in several ways. Prior literature has considered the ways in which science and scientists are inherently focused on topics of interest to themselves which is a form of bias [4,5,7]. This essentially impedes the ability for value-free knowledge production. By examining how researchers in a discipline – in this case, addiction and pain science – define important research agendas and evaluate their proposed innovations, we can identify topics or phenomenon that these scientists deem as important to evaluate and address. In our study, consistent themes of addictive pain treatments, overdose, and treatment development suggest that the framing of research agendas is likely reflective of public and professional discourse on public health crises and urgent unmet needs [38]. Additionally, a growing focus on how social problems particularly affect special (often vulnerable) populations suggests an emerging disciplinary focus on social determinants of health. Interestingly, though, most investigators who focused on predictors of addiction and pain focused on genetic factors and not socioenvironmental influences which indicates a preference to genetic over social influences. An examination of pain/addiction conceptualization also offers insight into how authors understand these issues. We also found that researchers were most often evaluating prescription drugs as treatments for various addiction/pain conditions, highlighting the discipline’s preference for medication treatments over behavioral therapies or alternative innovations – though the scope of proposed treatments does reflect growing willingness to pursue other methods. Moreover, the emphasis on efficacy over other implementation or population effectiveness measures lends insight into how researchers prioritize important study outcomes.

This study advances existing research on pain and addiction. Prior research has been limited due to deductive approaches and analysis of select published papers, which biases the identified research trends. This inductive approach and our analysis of study abstracts funded by one of the leading grant-funding agencies offers a closer look at what topics researchers’ study and what topics NIH-HEAL funds. Given these clear emerged patterns, our analysis of NIH-HEAL funded studies suggests that researchers are focused on specific, common issues and innovations, which are likely influenced by societal and scientific discourse as well as a researcher’s own area of interest. Given their decision to fund these studies, NIH-HEAL appears to favor these lines of inquiry over others.

### Contribution to practice

Our work carries implications for scientists, grant-funding agencies, and treatment consumers. For scientists, our findings call for researchers to consider the rationale for the studies, the state of science, and the needs of practitioners/the community in terms of using the scientific products. For grant-funding agencies, if organizations are interested in broadening their funded research portfolio, they may consider urging scientists to pursue non-traditional topics and innovation approaches/evaluations. Finally, for treatment consumers, our work suggests that scientists focus primarily on evaluating the efficacy of prescription drugs, which in practice limits people’s menu of treatment options for any given condition. Moreover, necessary policies and/or treatments could be inappropriate or even nonexistent given a lack of scientific attention to a topic or treatment.

A recent research trend is to include researcher-practitioner partnerships (e.g., providers, justice actors, school administrators, etc.) and those with lived experience (e.g., patients, families, community members, etc.) in the research process. These partnerships are useful to help researchers move towards efforts to improve clinical practice for a variety of subpopulations and in a variety of settings [6]. Including both practitioner and patient voices in the research process may generate new topics for research, as well as ensure the “on the ground” issues guide the research questions [39]. By having practitioners, patients, and researchers work together towards addressing critical field issues, this approach aims to address some of the concerns about the gaps in terms of research topics, outcomes, and impact of the science, including objectivity. The challenge will be whether funding agencies will view these priorities as important. NIH-HEAL has demonstrated a commitment to this trend, writing in one online post, “patient engagement and its transformative impact on research and health care was on the minds of all of us early on… Stakeholders, patients and families, and advocates were center stage” [40]. The establishment of the Native Collective Research Effort to Enhance Wellness [41] is one example of NIH-HEAL’s enduring commitment to inclusive and culturally competent research and practice. However, due to the investigator-initiated funding model used by NIH-HEAL, partnership decisions may be ultimately left up to the investigator(s) or a product of specific funding announcements that require partnerships like the Clinical Trials Network, Justice Community Opioid Innovation Network, and HEALing Communities. Our landscape analysis revealed that only 162 studies considered stakeholder needs and 265 studies consulted individuals with lived experience to inform innovation designs – indicating a lack of provider and patient engagement in the HEAL portfolio. Moreover, while the funded portfolio is indeed broad, our finding that researchers prefer treating addiction and pain conditions with prescription medication and have a siloed focus on efficacy may affect the ability to achieve the stated goals of inclusive and culturally competent research and practice. Mandated researcher-practitioner partnerships and inclusion of lived experience may be one remedy to this issue.

### Limitations and future research

There are two notable limitations. First, abstracts from the NIH RePORTER website were the primary material reviewed for this research, and concept papers or other material were only reviewed when they were available. This may create an inaccuracy in the numbers and data presented here because investigators could not have mentioned a component of their research in the abstract. Abstracts are also usually focused on the innovation and less so on the project methodology, which might bias the “outcome” findings presented here. Second, to create the motivation and outcome scripts, team members used Qualtrics to extract information from each abstract and then these scripts were linked to Atlas.ti and coded. During coding, when scripts were missing a substantial amount of information, NIH RePORTER was reviewed to add more context/codes. However, because of this, not all of the abstracts were reviewed for a second time during the qualitative coding, meaning that some information may not have been recorded.

Future research could expand upon this analysis in several interesting ways. First, it could be informative to explore this article’s research question using a quantitative approach, perhaps using regression techniques to identify predictors of investigators motivations and outcomes, or through latent class and/or cluster analysis to sort investigators into case membership based on their engagement with certain indicators. Researchers could also use the framework and techniques presented here to analyze investigator motivations/outcomes in other funding agencies or other fields. Finally, in-depth interviews and/or focus groups with researchers could yield additional information into why they identified and conceptualized social problems in certain ways, why they pursued specific solutions and/or study areas, and why they chose their proposed innovations and outcome measures. This research would improve understanding of how investigators decide what constitutes important knowledge production and the factors that may underlie these choices.

## Conclusions

This analysis sought to understand how authors funded by the NIH-HEAL initiative identify and conceptualize social problems, the solutions or study areas that authors note are necessary to alleviate identified problems, and the different ways that authors evaluate their proposed treatments and innovations. Through a directed content analysis of 1,068 NIH-HEAL funded study abstracts, we found that while the scope of NIH-HEAL funded studies is broad, patterns exist within the funded research. Investigators consistently focused on issues like addictive pain treatments, overdose, and treatment development, and there was a notable focus on how social problems particularly impact special (often vulnerable) populations. Investigators were also interested in genetic predictors of pain and addiction. By reviewing how authors conceptualize pain and addiction, we are better able to ascertain how scholars understand the lived experience of pain and addiction like impacts on wellness and perceptions of treatment. Finally, while authors proposed a variety of treatment innovations, most focused on prescription drugs, and most evaluation outcomes focused on efficacy. This research carries important implications for knowledge development, grant-funding agencies, scientists, and patients. Specifically, although the funded portfolio is diverse, our findings indicate that researchers tend to prioritize treating addiction and pain conditions with prescription medication and focus narrowly on efficacy. This suggests that NIH-HEAL may fall short of its stated objectives of promoting inclusive and culturally competent research and practice. In the future, mandating researcher-practitioner partnerships and incorporating people with lived experience could help address this concern.

## Supporting information

S1 File Data Study. (DOCX)
