# Supplementary material for: Shaping science: Scholarly motivation and research outcomes in NIH-HEAL funded studies
Source: PLoS One. 2026 Mar 3;21(3):e0343417. doi: 10.1371/journal.pone.0343417 (PMC12956126; doi:10.1371/journal.pone.0343417)
Supplement: S1 File — (DOCX) [file pone.0343417.s001.docx]

**Data Availability**

Our research team analyzed 1,072 research abstracts that are publicly available on the NIH RePORTER website (https://reporter.nih.gov/). For a list of the 1,072 study ID numbers, please see below:

| **Study ID Number** |
| --- |
| 9444707 |
| 9459809 |
| 9519355 |
| 9524666 |
| 9530717 |
| 9545385 |
| 9552022 |
| 9555046 |
| 9585836 |
| 9599337 |
| 9608089 |
| 9608532 |
| 9608544 |
| 9613911 |
| 9613939 |
| 9614028 |
| 9621132 |
| 9621475 |
| 9621873 |
| 9622261 |
| 9622295 |
| 9653772 |
| 9660796 |
| 9661530 |
| 9674194 |
| 9674683 |
| 9675407 |
| 9676620 |
| 9676787 |
| 9676886 |
| 9676930 |
| 9677008 |
| 9677597 |
| 9678382 |
| 9679778 |
| 9680193 |
| 9680488 |
| 9680910 |
| 9681118 |
| 9683950 |
| 9684195 |
| 9706434 |
| 9707184 |
| 9707258 |
| 9709506 |
| 9712327 |
| 9713551 |
| 9713716 |
| 9716866 |
| 9719019 |
| 9728195 |
| 9729182 |
| 9731951 |
| 9734921 |
| 9735591 |
| 9736138 |
| 9736358 |
| 9738472 |
| 9739061 |
| 9739674 |
| 9740089 |
| 9740762 |
| 9742720 |
| 9743019 |
| 9743427 |
| 9743491 |
| 9743910 |
| 9743912 |
| 9745741 |
| 9745990 |
| 9746820 |
| 9748221 |
| 9748222 |
| 9748228 |
| 9748729 |
| 9748773 |
| 9750304 |
| 9751487 |
| 9751502 |
| 9752121 |
| 9752189 |
| 9752205 |
| 9752598 |
| 9752844 |
| 9755001 |
| 9755186 |
| 9755187 |
| 9755668 |
| 9757837 |
| 9761676 |
| 9761823 |
| 9763068 |
| 9765878 |
| 9767352 |
| 9769432 |
| 9770033 |
| 9770072 |
| 9770076 |
| 9770483 |
| 9770484 |
| 9770485 |
| 9771125 |
| 9772615 |
| 9775470 |
| 9775669 |
| 9775762 |
| 9776687 |
| 9777758 |
| 9778341 |
| 9778453 |
| 9781338 |
| 9782089 |
| 9784924 |
| 9786265 |
| 9794170 |
| 9796231 |
| 9796252 |
| 9796632 |
| 9806827 |
| 9814892 |
| 9815836 |
| 9816140 |
| 9816362 |
| 9816476 |
| 9816498 |
| 9816541 |
| 9821308 |
| 9821520 |
| 9823898 |
| 9827054 |
| 9827701 |
| 9827922 |
| 9828185 |
| 9828246 |
| 9828948 |
| 9829265 |
| 9829469 |
| 9829475 |
| 9829976 |
| 9830397 |
| 9830874 |
| 9832226 |
| 9833817 |
| 9836991 |
| 9837108 |
| 9837181 |
| 9837189 |
| 9837724 |
| 9837725 |
| 9838457 |
| 9838598 |
| 9839124 |
| 9839232 |
| 9839289 |
| 9839338 |
| 9841017 |
| 9843839 |
| 9844337 |
| 9845104 |
| 9845353 |
| 9845965 |
| 9846177 |
| 9846812 |
| 9846901 |
| 9847335 |
| 9847817 |
| 9848023 |
| 9848027 |
| 9848085 |
| 9848092 |
| 9848313 |
| 9848378 |
| 9848425 |
| 9848902 |
| 9849933 |
| 9850412 |
| 9850643 |
| 9852022 |
| 9852791 |
| 9855321 |
| 9856648 |
| 9856898 |
| 9857109 |
| 9857255 |
| 9857901 |
| 9857979 |
| 9858490 |
| 9860339 |
| 9860408 |
| 9860960 |
| 9862038 |
| 9864857 |
| 9866221 |
| 9867358 |
| 9867952 |
| 9868113 |
| 9868114 |
| 9868133 |
| 9868758 |
| 9868783 |
| 9869148 |
| 9869164 |
| 9869461 |
| 9869481 |
| 9869574 |
| 9869813 |
| 9870024 |
| 9870070 |
| 9870237 |
| 9870482 |
| 9873216 |
| 9876499 |
| 9877133 |
| 9879277 |
| 9881018 |
| 9881407 |
| 9881988 |
| 9882011 |
| 9882365 |
| 9882422 |
| 9882650 |
| 9882676 |
| 9882684 |
| 9882727 |
| 9882751 |
| 9882789 |
| 9882805 |
| 9882806 |
| 9882807 |
| 9882808 |
| 9882809 |
| 9882827 |
| 9882828 |
| 9882829 |
| 9882831 |
| 9882832 |
| 9882833 |
| 9882834 |
| 9882847 |
| 9882856 |
| 9888546 |
| 9888733 |
| 9888821 |
| 9888896 |
| 9889726 |
| 9891326 |
| 9891612 |
| 9892107 |
| 9893166 |
| 9894581 |
| 9896660 |
| 9897398 |
| 9897962 |
| 9897963 |
| 9897965 |
| 9897966 |
| 9897967 |
| 9897968 |
| 9897969 |
| 9898044 |
| 9898052 |
| 9898100 |
| 9898106 |
| 9898107 |
| 9898108 |
| 9898109 |
| 9898110 |
| 9898111 |
| 9898112 |
| 9898132 |
| 9898133 |
| 9898134 |
| 9898135 |
| 9898136 |
| 9898137 |
| 9898138 |
| 9898139 |
| 9898607 |
| 9898661 |
| 9898687 |
| 9898784 |
| 9898919 |
| 9899070 |
| 9899405 |
| 9899464 |
| 9899486 |
| 9899532 |
| 9899542 |
| 9899554 |
| 9899660 |
| 9900193 |
| 9900195 |
| 9900227 |
| 9900231 |
| 9900258 |
| 9900280 |
| 9900281 |
| 9900284 |
| 9900287 |
| 9900304 |
| 9900325 |
| 9900350 |
| 9900353 |
| 9900356 |
| 9900364 |
| 9900385 |
| 9900395 |
| 9900424 |
| 9900443 |
| 9900516 |
| 9900529 |
| 9900560 |
| 9901704 |
| 9901764 |
| 9901871 |
| 9901874 |
| 9901875 |
| 9901876 |
| 9901877 |
| 9901878 |
| 9901879 |
| 9901880 |
| 9902068 |
| 9902084 |
| 9902131 |
| 9902582 |
| 9902764 |
| 9903903 |
| 9904032 |
| 9904355 |
| 9904461 |
| 9905066 |
| 9905069 |
| 9905086 |
| 9905170 |
| 9905182 |
| 9905262 |
| 9905308 |
| 9905430 |
| 9906080 |
| 9906345 |
| 9907592 |
| 9907601 |
| 9908492 |
| 9908597 |
| 9908680 |
| 9908734 |
| 9909162 |
| 9909345 |
| 9909401 |
| 9910282 |
| 9911512 |
| 9912034 |
| 9912257 |
| 9912345 |
| 9912548 |
| 9912559 |
| 9912595 |
| 9912651 |
| 9913686 |
| 9913806 |
| 9917204 |
| 9919033 |
| 9927826 |
| 9932691 |
| 9932702 |
| 9932733 |
| 9932739 |
| 9932778 |
| 9932780 |
| 9939241 |
| 9939270 |
| 9939753 |
| 9940239 |
| 9940967 |
| 9941765 |
| 9942319 |
| 9944015 |
| 9950853 |
| 9954486 |
| 9954760 |
| 9961929 |
| 9963442 |
| 9966115 |
| 9966143 |
| 9966187 |
| 9968676 |
| 9972347 |
| 9974791 |
| 9974863 |
| 9974866 |
| 9974940 |
| 9976792 |
| 9979208 |
| 9980049 |
| 9980635 |
| 9981089 |
| 9982460 |
| 9982464 |
| 9983431 |
| 9987897 |
| 9992422 |
| 9993727 |
| 9993931 |
| 9994052 |
| 9994053 |
| 9994056 |
| 9997567 |
| 9998095 |
| 9998189 |
| 10000668 |
| 10004917 |
| 10010119 |
| 10013066 |
| 10013399 |
| 10015866 |
| 10017371 |
| 10020453 |
| 10020661 |
| 10022491 |
| 10026953 |
| 10054792 |
| 10055369 |
| 10055490 |
| 10055582 |
| 10056337 |
| 10057199 |
| 10058548 |
| 10078437 |
| 10082913 |
| 10085851 |
| 10086831 |
| 10113197 |
| 10113291 |
| 10113419 |
| 10118262 |
| 10120176 |
| 10120215 |
| 10127813 |
| 10130067 |
| 10130155 |
| 10130233 |
| 10130863 |
| 10133699 |
| 10134612 |
| 10136935 |
| 10138162 |
| 10138638 |
| 10139200 |
| 10139426 |
| 10140502 |
| 10140552 |
| 10140834 |
| 10140939 |
| 10141532 |
| 10144277 |
| 10146081 |
| 10146999 |
| 10148091 |
| 10149170 |
| 10149450 |
| 10151404 |
| 10151730 |
| 10152003 |
| 10153043 |
| 10154341 |
| 10155301 |
| 10155895 |
| 10156633 |
| 10157227 |
| 10157937 |
| 10157953 |
| 10161464 |
| 10163016 |
| 10163408 |
| 10164520 |
| 10164521 |
| 10164974 |
| 10166251 |
| 10167308 |
| 10167785 |
| 10168176 |
| 10168769 |
| 10169854 |
| 10170518 |
| 10170530 |
| 10173220 |
| 10173503 |
| 10174525 |
| 10175416 |
| 10175561 |
| 10175594 |
| 10175741 |
| 10176122 |
| 10176852 |
| 10177030 |
| 10177211 |
| 10177229 |
| 10178740 |
| 10186825 |
| 10188053 |
| 10197802 |
| 10197811 |
| 10199299 |
| 10200231 |
| 10200501 |
| 10202826 |
| 10204264 |
| 10208052 |
| 10208059 |
| 10208162 |
| 10208316 |
| 10208334 |
| 10208515 |
| 10209032 |
| 10209094 |
| 10210513 |
| 10212539 |
| 10214765 |
| 10214771 |
| 10216519 |
| 10216546 |
| 10219462 |
| 10219647 |
| 10223026 |
| 10223795 |
| 10224388 |
| 10224964 |
| 10231558 |
| 10233551 |
| 10236607 |
| 10236683 |
| 10239279 |
| 10239919 |
| 10245396 |
| 10250448 |
| 10250682 |
| 10250727 |
| 10253179 |
| 10253306 |
| 10253377 |
| 10255305 |
| 10258492 |
| 10258730 |
| 10259365 |
| 10259366 |
| 10259367 |
| 10259369 |
| 10259387 |
| 10259507 |
| 10261709 |
| 10262845 |
| 10263436 |
| 10264296 |
| 10264717 |
| 10267445 |
| 10267804 |
| 10282421 |
| 10286333 |
| 10286886 |
| 10291187 |
| 10303378 |
| 10304029 |
| 10304570 |
| 10304647 |
| 10318547 |
| 10320676 |
| 10321502 |
| 10321798 |
| 10322523 |
| 10322644 |
| 10326436 |
| 10326595 |
| 10326651 |
| 10328176 |
| 10329053 |
| 10329705 |
| 10329804 |
| 10331194 |
| 10333110 |
| 10348860 |
| 10351460 |
| 10351640 |
| 10351973 |
| 10352954 |
| 10354615 |
| 10358798 |
| 10364578 |
| 10372486 |
| 10373400 |
| 10373461 |
| 10375955 |
| 10375971 |
| 10375975 |
| 10375976 |
| 10375977 |
| 10375979 |
| 10376890 |
| 10377726 |
| 10378274 |
| 10378283 |
| 10378328 |
| 10378402 |
| 10378422 |
| 10378786 |
| 10378875 |
| 10378902 |
| 10378910 |
| 10378922 |
| 10378923 |
| 10378942 |
| 10378952 |
| 10378969 |
| 10378979 |
| 10378982 |
| 10379584 |
| 10379694 |
| 10379705 |
| 10380197 |
| 10380355 |
| 10380359 |
| 10380398 |
| 10380426 |
| 10380522 |
| 10381046 |
| 10381103 |
| 10381109 |
| 10382876 |
| 10385311 |
| 10386341 |
| 10386456 |
| 10387104 |
| 10387137 |
| 10390149 |
| 10390733 |
| 10391075 |
| 10391263 |
| 10395664 |
| 10397259 |
| 10398329 |
| 10398386 |
| 10398387 |
| 10398388 |
| 10398389 |
| 10398390 |
| 10398391 |
| 10398392 |
| 10398393 |
| 10398444 |
| 10398527 |
| 10398627 |
| 10399783 |
| 10399813 |
| 10399907 |
| 10400307 |
| 10400321 |
| 10400340 |
| 10400379 |
| 10400445 |
| 10400459 |
| 10400468 |
| 10400476 |
| 10400589 |
| 10403058 |
| 10403871 |
| 10404154 |
| 10405235 |
| 10406064 |
| 10408897 |
| 10413418 |
| 10414223 |
| 10415416 |
| 10415626 |
| 10419922 |
| 10426755 |
| 10426787 |
| 10428343 |
| 10429456 |
| 10434255 |
| 10434632 |
| 10435316 |
| 10436556 |
| 10438018 |
| 10439270 |
| 10440189 |
| 10441666 |
| 10441986 |
| 10442798 |
| 10450294 |
| 10450295 |
| 10450923 |
| 10453929 |
| 10453930 |
| 10454012 |
| 10454583 |
| 10455129 |
| 10456354 |
| 10456452 |
| 10460178 |
| 10462315 |
| 10462326 |
| 10467167 |
| 10468273 |
| 10471577 |
| 10473047 |
| 10474683 |
| 10476034 |
| 10476678 |
| 10476705 |
| 10477526 |
| 10478324 |
| 10479208 |
| 10479425 |
| 10481923 |
| 10482203 |
| 10485104 |
| 10485389 |
| 10485408 |
| 10485444 |
| 10485580 |
| 10485642 |
| 10485805 |
| 10485915 |
| 10485920 |
| 10486286 |
| 10488140 |
| 10490956 |
| 10494199 |
| 10494299 |
| 10499934 |
| 10505802 |
| 10507724 |
| 10507932 |
| 10508272 |
| 10510245 |
| 10511349 |
| 10512191 |
| 10512217 |
| 10512599 |
| 10512672 |
| 10512706 |
| 10512708 |
| 10517225 |
| 10518516 |
| 10519404 |
| 10519880 |
| 10520639 |
| 10521941 |
| 10523190 |
| 10526631 |
| 10531766 |
| 10532462 |
| 10535154 |
| 10536153 |
| 10536567 |
| 10537743 |
| 10538202 |
| 10538983 |
| 10539159 |
| 10539166 |
| 10544440 |
| 10546418 |
| 10547922 |
| 10547925 |
| 10552492 |
| 10558270 |
| 10569208 |
| 10569720 |
| 10569775 |
| 10569868 |
| 10571306 |
| 10571425 |
| 10571508 |
| 10572041 |
| 10573954 |
| 10574306 |
| 10578063 |
| 10578869 |
| 10579668 |
| 10580155 |
| 10580406 |
| 10580415 |
| 10580477 |
| 10580905 |
| 10580923 |
| 10580933 |
| 10581160 |
| 10581282 |
| 10581405 |
| 10582847 |
| 10582989 |
| 10583133 |
| 10583271 |
| 10583339 |
| 10584684 |
| 10587283 |
| 10587594 |
| 10587698 |
| 10588405 |
| 10588501 |
| 10588504 |
| 10588517 |
| 10588669 |
| 10588672 |
| 10588805 |
| 10588855 |
| 10588908 |
| 10589388 |
| 10589466 |
| 10589481 |
| 10589518 |
| 10589601 |
| 10589995 |
| 10590000 |
| 10590013 |
| 10590040 |
| 10590120 |
| 10590166 |
| 10590167 |
| 10590186 |
| 10590209 |
| 10590218 |
| 10590236 |
| 10590246 |
| 10590267 |
| 10590281 |
| 10590299 |
| 10590303 |
| 10590310 |
| 10590322 |
| 10590364 |
| 10590378 |
| 10590442 |
| 10590443 |
| 10590474 |
| 10590481 |
| 10590541 |
| 10591779 |
| 10592520 |
| 10592780 |
| 10593656 |
| 10593657 |
| 10593658 |
| 10593659 |
| 10593660 |
| 10593661 |
| 10593843 |
| 10593844 |
| 10593845 |
| 10593846 |
| 10593847 |
| 10593848 |
| 10593849 |
| 10594334 |
| 10594335 |
| 10594336 |
| 10594337 |
| 10594338 |
| 10594339 |
| 10594340 |
| 10594341 |
| 10594594 |
| 10595133 |
| 10595357 |
| 10595421 |
| 10595896 |
| 10595974 |
| 10596435 |
| 10596436 |
| 10596437 |
| 10596438 |
| 10596439 |
| 10596851 |
| 10596875 |
| 10597344 |
| 10597790 |
| 10597861 |
| 10598312 |
| 10598724 |
| 10598873 |
| 10599385 |
| 10599396 |
| 10599401 |
| 10599675 |
| 10601192 |
| 10602826 |
| 10603008 |
| 10607479 |
| 10608279 |
| 10608403 |
| 10608491 |
| 10608851 |
| 10614222 |
| 10614763 |
| 10615366 |
| 10615519 |
| 10616580 |
| 10616932 |
| 10617101 |
| 10617997 |
| 10618645 |
| 10619029 |
| 10621059 |
| 10622257 |
| 10622997 |
| 10628516 |
| 10628540 |
| 10635746 |
| 10638278 |
| 10638816 |
| 10641500 |
| 10642506 |
| 10642544 |
| 10645536 |
| 10646600 |
| 10646991 |
| 10650569 |
| 10650650 |
| 10652027 |
| 10655111 |
| 10655675 |
| 10655828 |
| 10663762 |
| 10667038 |
| 10667141 |
| 10667277 |
| 10673373 |
| 10681111 |
| 10683816 |
| 10683849 |
| 10684399 |
| 10684486 |
| 10684487 |
| 10684501 |
| 10684558 |
| 10685784 |
| 10686688 |
| 10688445 |
| 10689000 |
| 10690167 |
| 10691962 |
| 10692456 |
| 10692504 |
| 10694574 |
| 10694726 |
| 10695275 |
| 10695542 |
| 10695795 |
| 10696574 |
| 10696992 |
| 10701506 |
| 10701510 |
| 10701528 |
| 10705887 |
| 10706844 |
| 10709410 |
| 10710264 |
| 10715903 |
| 10717184 |
| 10721763 |
| 10722105 |
| 10722768 |
| 10722943 |
| 10724026 |
| 10726405 |
| 10726834 |
| 10730831 |
| 10738693 |
| 10739074 |
| 10740629 |
| 10740793 |
| 10740796 |
| 10741388 |
| 10741606 |
| 10744961 |
| 10745058 |
| 10745106 |
| 10745471 |
| 10745526 |
| 10745607 |
| 10745632 |
| 10747239 |
| 10747646 |
| 10747661 |
| 10748148 |
| 10748225 |
| 10748273 |
| 10748428 |
| 10748467 |
| 10748567 |
| 10748626 |
| 10748634 |
| 10749222 |
| 10749358 |
| 10750077 |
| 10750125 |
| 10750254 |
| 10750306 |
| 10750458 |
| 10750475 |
| 10750480 |
| 10750770 |
| 10753356 |
| 10757180 |
| 10758937 |
| 10759091 |
| 10759100 |
| 10759550 |
| 10759735 |
| 10760487 |
| 10761260 |
| 10765049 |
| 10765181 |
| 10765272 |
| 10765299 |
| 10771064 |
| 10771803 |
| 10771904 |
| 10772463 |
| 10772665 |
| 10772818 |
| 10773680 |
| 10774377 |
| 10774464 |
| 10774563 |
| 10774593 |
| 10775030 |
| 10775120 |
| 10775216 |
| 10775250 |
| 10775597 |
| 10776106 |
| 10777818 |
| 10777825 |
| 10783274 |
| 10783393 |
| 10783610 |
| 10783888 |
| 10784019 |
| 10784207 |
| 10784209 |
| 10784407 |
| 10785110 |
| 10785873 |
| 10786660 |
| 10789054 |
| 10794463 |
| 10794609 |
| 10794761 |
| 10794862 |
| 10805071 |
| 10805159 |
| 10806545 |
| 10806601 |
| 10809150 |
| 10810953 |
| 10812062 |
| 10812139 |
| 10812628 |
| 10812631 |
| 10812703 |
| 10812747 |
| 10812798 |
| 10812813 |
| 10812890 |
| 10813313 |
| 10819946 |
| 10822612 |
| 10822921 |
| 10823599 |
| 10831206 |
| 10878304 |
